# Supplementary material for: Sex differences in self-perceived employability and self-motivated strategies for learning in Polish first-year students
Source: PLoS One. 2022 May 13;17(5):e0264817. doi: 10.1371/journal.pone.0264817 (PMC9106146; doi:10.1371/journal.pone.0264817)
Supplement: Attachment — Submitted filename: 3. PONE-D-21-29167- Reviewer Comments.docx [file pone.0264817.s001.docx]

**PONE-D-21-29167: The relationship between self - motivated strategies for learning and self-perceived employability in the first-year Polish male and female students**

*The paper aims at searching the connections between females and males’ self-motivated strategies for learning (MSQL) and employability of university students (n=600) in a Central European context. Analyses revealed significant differences in the patterns of correlation between male and female’s MSQL and employability (both internal and external). Females, despite their higher results in MSQL presented lower internal employability and general self-efficacy*. According to the authors, their *results will provide evidence to support HEI curricular development and workplaces attitude change to minimize existing sex inequalities.*

Below are my comments for possible revision.

**Major Points**

**Results**

The authors tested 10 hypotheses in total- H1 to H7 were tested using the Independent t-test, while H8 to H10 were tested using Pearson’s Correlation. However, the authors go on to present an Intercorrelation of SPE and MLSD Factors in males and females. What is the rationale for doing this?

Also, what is the rationale for correlating MSLQ general with MSLQ factors? Certainly the correlations will be high because each of these factors contribute to the MSLQ general score. The same applies to the Intercorrelation of SPE.

Again what is the justification for the ‘sex difference in intercorrelations of MSLQ factors if the authors compared gender differences on the various variables using a t-test?

Three hypothesis, H8 to 10 predicted a relationship between specific variables and yet, the authors chose to correlate all their variable of study. Why did they not focus on the three hypothesis that had been stated? This makes it seem as though they are merely ‘fishing’ for results and therefore just decided to run as many analysis as they could to be able to generate several results.

***Discussion***

There is very scanty information on the discussion on H8 to H10. A lot more could have been said to explain the findings. Generally, most of the findings are not well discussed. How will the author explain their own findings and what do these findings mean?

Seven hypotheses are tested to compare males and females on several variables. There are 5 hypotheses which females scored higher than males but these are not discussed. Instead, the whole focus of the Discussion and Implications is on, and centered around the results of the two hypothesis where males scored higher than females. The question is why do the authors ignore all of these other results and just focus on the findings of 2 out of 10 hypotheses?

Generally, the focus of the Discussion seems to be all about how females presented low internal employability and general self-efficacy I find the discussion to be very biased and skewed because there were several other variables that females scored higher than males but these not discussed.

Page 10 Line 304- 310: *Our results indicated that women’s mental barriers might be worse than any institutional barriers to their career advancement*. *Hence, there is a rising need to provide female students with psychological assistance. In the case when a person experiences learned helplessness, reflected in a sense of decreased efficiency, an appropriate intervention may involve exploring coping strategies. It is crucial to include in the higher education curriculum discussions about the psychological determinants of the underrepresentation of women in managerial positions rather than structural barriers.*

What is the above assertion in reference to? Which of the study variable assessed, and which finding is it in reference to? I think this is far-fetched!

**Implications**

The ‘implications’ of the study is lengthier than the Discussion of results and I find that quite unusual. Again there are so many findings of several hypotheses that were confirmed in favour of females which the authors ignore.

**Limitations**

The authors do not discuss any limitations of the study. Note that every study has some limitations.

**Minor Points**

**Introduction**

***SPE’s factors***

Page 2 Line 32: *External SPE factors include characteristic features of a local labour market and specific qualifications.* The authors should list what some of these *characteristic features of a local labour marke*t are.

Line 88- *Hence, in our study, we predicted that Polish first-year female students also*

*outperformed males in the case of learning strategies (H2).* Use the correct tense.

The abbreviation EMP is not written in full in any part of the paper. Please specify what this stands for.

**Method**

***Subject***

It will be worth explaining how the 600 students were recruited, what sampling technique was used etc. and details about the participants such as examples of their subject of study.

The term ‘Participants’ should replace ‘Subjects’ as that is the more acceptable convention.

***Polish adaptation of the self-perceived Employability Scale***

Page 4 Line 130- *-* the authors indicates 6 items on the scale but yet lists a total of 8 items consisting of 3 *internal* and 5 *external* items.

***The MSLQ***

Page 4 Line 150: *We considered our sample as learners whose approach to learning is still closer to school students than university students.* What does *closer to school students* mean?

***Procedure and ethical consideration***

The authors should specify the name of the ethics committee where approval was obtained.

**Best wishes.**
